# Chemical Peels in Skin of Color: A Scoping Review of Safety, Efficacy, and Practice Patterns

**DOI:** 10.7759/cureus.108851

**Published:** 2026-05-14

**Authors:** Emily Garelick, Priya Pohani, Anjiya Aswani, Sophia Khan, Saloni Chadha, Tej Patel, Mohamad Bilal Kebbe

**Affiliations:** 1 Dermatology, Philadelphia College of Osteopathic Medicine, Philadelphia, USA; 2 Dermatology, Alabama College of Osteopathic Medicine, Dothan, USA; 3 Internal Medicine, Ross University School of Medicine, Bridgetown, BRB; 4 Dermatology, Washington University of Health and Science, San Pedro, BLZ; 5 Medicine, Lake Erie College of Osteopathic Medicine, Bradenton, USA; 6 Medicine, Lewis Katz School of Medicine, Philadelphia, USA; 7 Dermatology, Okan University Faculty of Medicine, Istanbul, TUR

**Keywords:** chemical peels, fitzpatrick skin phototypes iv-vi, glycolic acid, skin of color, trichloroacetic acid

## Abstract

Chemical peels are widely used for both cosmetic and medical dermatologic indications, but their application in individuals with skin of color (Fitzpatrick skin types IV-VI) requires caution due to the increased risk of pigmentary complications. To assess their safety and efficacy, a comprehensive PubMed review was conducted, identifying 473 studies, seven of which met the inclusion criteria, specifically those articles documenting chemical peels in skin of color populations and reported primary outcomes. Among the seven included studies, five evaluated peels for the treatment of acne and acne scarring, all reporting positive outcomes with minimal adverse effects. One study addressed lichen planus pigmentosus, showing significant clinical improvement, and one highlighted chemical burns resulting from improper use. Overall, superficial peels such as glycolic and salicylic acid show promise for acne and pigmentary disorders with good safety profiles. However, research is limited by small sample sizes, short follow-ups, and underrepresentation of Fitzpatrick skin types IV-VI. Further research is needed to establish standardized protocols, assess deeper peels, and guide equitable, evidence-based care.

## Introduction and background

Chemical peels are a commonly used method of chemoexfoliation that involves applying a chemical agent to the skin, which intentionally induces controlled injury and the subsequent regeneration of the skin [1]. These peels serve as both cosmetic and therapeutic and are commonly used to improve the texture and status of the skin. This directly aids in improving conditions like acne and even actinic keratoses, which are rough and scaly precancer spots found on the skin [2]. Chemical peels are used to smooth the skin surface, reducing the evidence of wrinkles, creating a more youthful appearance [3]. The potency of the chemical peel is influenced by the pKa of its active ingredient [1]. A lower pKa corresponds to a stronger acid and therefore a more potent exfoliant [1,4]. Keratolytic chemical peels may contain glycolic acid, salicylic acid, mandelic acid, lactic acid, pyruvic acid, and retinoic acid, which help to break down bonds between skin cells [1,2]. Together, the keratolysis and protein denaturation during a chemical peel treatment set a cascade of skin healing leading to collagen and elastin deposition [5]. Typically, several weeks before a chemical peel, a priming agent is applied to the skin to gently thin the stratum corneum, the outermost layer, thereby enhancing the peel's effectiveness as it is likely to penetrate the skin more efficiently [1]. During the procedure, careful consideration of eye protection and skin cleansing is important [5]. Post-treatment, patients may experience peeling and intense redness of the skin, which takes a few weeks to subside, depending on the strength and ingredients used in the treatment [1]. Clinicians should also educate patients on proper sunscreen application for ultraviolet protection before and after the chemical peel, along with recommending gentle cleansers, emollients, and the use of ice packs to minimize inflammation.

Chemical peels are generally classified into three categories based on their depth of skin penetration: superficial, medium, and deep [6,7]. In skin of color, especially Fitzpatrick skin types (FST) IV-VI, all categories of chemical peels must be carefully performed, as there is an increased risk of scarring and altered pigment [8]. FST IV is characterized by individuals with olive or light brown skin tones, who tan more easily than average and rarely experience sunburn. FST V is characterized by individuals with brown or dark brown skin who tan very easily and seldom develop sunburn [8]. FST VI is characterized by individuals with black skin who rarely experience sunburn and tan effortlessly [1,9]. While chemical peels that reach the melanin-producing layers of the skin can be used to even out hyperpigmentation, they also carry a higher risk of complications in these skin types, including hypertrophic scars and keloid formation [10]. Post-inflammatory hyperpigmentation (PIH) is another important consideration in patients with skin of color, which is one of the reasons that clinicians may be hesitant to use chemical peels on darker skin [10]. Additional clinical contraindications on any skin type may include open wounds, a history of allergic reactions, poor nutritional status, and lifestyle factors such as smoking, which may impair healing. Further research on these risks and hesitations may increase the safety and efficacy of exfoliation using chemical peels in all skin colors. Additionally, medical literature has insufficient data on the socioeconomic considerations of cosmetic therapies such as chemical peels, despite the increased prevalence and interest in ethnic groups [11]. Chemical peels are generally cost-effective and may be combined with other forms of cosmetic therapies including laser therapy; thus, there may be an added benefit in additional clinical trials on combination cosmetic therapies, especially in diverse skin colors [10].

Despite the growing demand for cosmetic procedures among patients with skin of color, much of the foundational chemical peel literature has historically centered on lighter skin types, with inconsistent stratification by FST and limited long-term safety data in FST IV-VI. This creates a clinically relevant evidence gap, as practitioners must balance therapeutic benefit against heightened risks of dyschromia and scarring in a population that is expanding and uniquely vulnerable to pigmentary sequelae. A comprehensive synthesis of available data is therefore needed to clarify which peel types, concentrations, and protocols demonstrate favorable safety and efficacy profiles specifically to skin of color.

A scoping review of chemical peel use in skin of color is needed, given the current lack of consolidated evidence and the increasing prevalence of chemical peels in practice. Patients with skin of color account for 32% of cosmetic procedures within the United States as of 2017, with the number only growing [9]. Current evidence shows the benefits of chemical peels for conditions that may cause dyschromia, such as melasma, acne, and PIH in individuals with skin of color [9]. Individuals pursuing chemical peel treatment are trending to be younger, in their third or fourth decades of life, which means that the effects of their chemical treatments will likely have a major effect on their lives. [8]. As younger patients receive these treatments, it is vital to evaluate their long-term efficacy and safety and how practitioners use chemical peels in practice.

Several intrinsic and extrinsic factors distinguish FST IV-VI from I-III, leading to distinct responses to chemical peels in patients with skin of color [12]. One of the defining intrinsic differences is the quantity of melanin pigment produced by melanocytes in darker skin types. Additionally, FST IV-VI have an increased quantity of elastic fibers, greater epidermal thickness, and smaller, but more highly reactive collagen [13]. These structural differences in the skin affect how skin of color reacts with the various chemicals used in chemical peels when compared to lighter skin tones that have been studied more [8,14]. Skin aging also differs between skin tones [13,15]. Literature shows that patients with FST IV-VI experience delayed photoaging due to the photoprotective properties of melanin and increased elastic properties of these individuals' skin [13]. Differences in perceived aging may lead some patients of color to seek out cosmetic treatments at later ages due to perceived less severe aging [16]. This could affect how the chemical peel may interact with their more mature skin. The differences in skin structure will also affect the type and depth of chemical peels that are safe to use in FST IV-VI without causing adverse effects [14]. 

This review aims to evaluate the effect of chemical peels on clinical outcomes, including efficacy and safety, in patients with skin of color (FST IV-VI) compared to alternative treatments or standard care. With this review, we aim to map and consolidate the available evidence evaluating the safety, efficacy, and practice patterns of chemical peels used in people with FST IV-VI.

## Review

Methods

Search Strategy

A structured literature search was conducted using the PubMed database on August 15, 2025. Predefined keywords and Boolean operators were applied to identify relevant studies examining the use of chemical peels in patients with skin of color. The complete search strategy is presented in Table 1.

**Table 1:** Search strategy used for literature retrieval

Database	Search date	Search string
PubMed	August 15, 2025	("Chemical Peel" OR "chemical peeling" OR "alpha hydroxy acid" OR "trichloroacetic acid" OR "glycolic acid" OR "salicylic acid") AND ("skin of color" OR "Fitzpatrick IV" OR "Fitzpatrick V" OR "Fitzpatrick VI" OR "African American" OR "Black" OR "Asian" OR "Hispanic")

**Figure 1:**
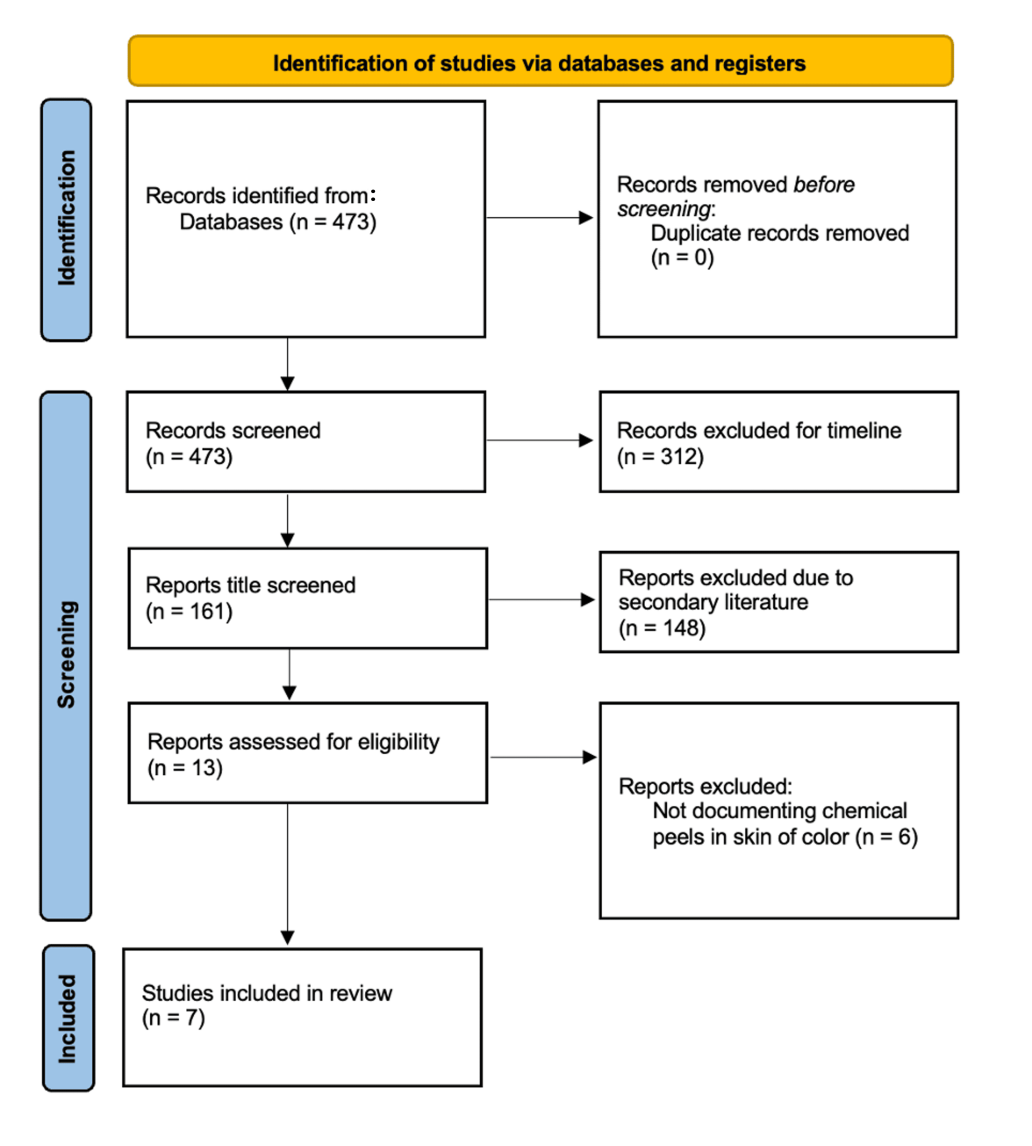
PRISMA-ScR flow diagram for scoping literature review on chemical peels in skin of color PRISMA-ScR: Preferred Reporting Items for Systematic Reviews and Meta-Analyses Extension for Scoping Reviews

The study selection process is illustrated in the Preferred Reporting Items for Systematic Reviews and Meta-Analyses Extension for Scoping Reviews (PRISMA-ScR) flow diagram (Figure 1).

Eligibility Criteria

The inclusion and exclusion criteria were defined a priori to ensure consistency and relevance of the selected studies. These criteria were developed in accordance with the PICOS framework, specifying the population, intervention, comparison, outcomes, and study designs of interest.

Studies were eligible for inclusion if they met all of the following criteria: (1) published between 2015 and 2025, (2) available as free full-text articles, (3) written in the English language, (4) included human subjects, (5) involved participants with skin of color, defined as individuals identifying as African American or Black, Hispanic or Latino, Asian, or Native American or Pacific Islander or those classified as FST IV, V, or VI, (6) evaluated the use of chemical peels, including but not limited to alpha-hydroxy acids, glycolic acid, salicylic acid, and trichloroacetic acid (TCA), and (7) reported clinical outcomes, safety profiles, adverse effects, or treatment efficacy related to chemical peels in skin of color.

Studies were excluded if they met any of the following criteria: (1) published prior to 2015, (2) not available as free full text, (3) non-English language publications, (4) animal studies or in vitro studies, (5) articles not specific to patients with skin of color or without stratified data applicable to this population, (6) studies evaluating cosmetic or dermatologic treatments other than chemical peels, (7) secondary sources, including narrative reviews, editorials, commentaries, expert opinions, conference abstracts, and letters to the editor, and (8) articles that did not align with the predefined inclusion criteria following full-text review.

These criteria guided the screening, eligibility assessment, and final selection of studies included in the scoping review.

Data Extraction

All identified records were screened independently by two reviewers (E.G. and P.P.) based on title and abstract, followed by full-text review for eligibility. Discrepancies between reviewers were resolved through discussion and consensus.

After screening and applying the inclusion criteria to the studies obtained from the relevant databases, researchers organized the information on a data log that included the title, type of review, and year, inclusion/exclusion criteria, sample size and age, diagnosis, treatment, and results. The final outcomes were documented on a Google Docs spreadsheet (Google Docs, Menlo Park, California, United States). With the information organized, a thorough discussion of each article was conducted to determine whether it fit the inclusion criteria.

The initial search elicited 473 articles that were screened based on the outlined search criteria. After removing 312, which were outside of the included date range and non-free full-text articles, an additional 148 were filtered out as they were secondary sources. Once the screening process was over, the remaining articles underwent a quality assessment process, whereby six articles that did not match the inclusion criteria were removed. The final articles selected involved the use of chemical peels for the treatment of acne, acne scarring, and lichen planus pigmentosus, as well as the dangers that misuse of chemical peels may cause in patients with skin of color.

Rationale for Narrative Scoping Review Approach

A narrative scoping methodology was selected rather than a formal meta-analysis due to the heterogeneity of the available literature. Existing studies evaluating chemical peels in skin of color vary substantially in study design, sample size, peel type and concentration, outcome measures, and duration of follow-up, limiting the feasibility of quantitative pooling. Additionally, many reports lack standardized stratification by FST or consistent reporting of pigmentary complications, further precluding robust comparative statistical synthesis. A scoping review, therefore, allows for broader mapping of the evidence landscape, identification of knowledge gaps, and clarification of practice patterns without imposing restrictive inclusion criteria required for meta-analytic methodology.

Results

Characteristics of the Included Studies

Seven studies are included in this scoping review (Table 2), ranging from 2015 to 2025, taking place in the United States, Iran, Switzerland, Thailand, China, South Korea, and Brazil. These studies include patients from FST I to VI. The peels used in these studies included the following ingredients: TCA, azelaic acid, salicylic acid, carbolic acid, glycolic acid, Jessner's solution, licochalcone A, 1,2-decanediol, L-carnitine, and other herbal substances. Five out of seven studies discussed acne and scarring, while one discussed chemical peels in the context of lichen planus pigmentosus, and the other documented a case report of a burn after using a chemical peel.

**Table 2:** Included articles following scoping review of the use of chemical peels in patients with skin of color FST: Fitzpatrick skin type

Authors	Study design	Peel type and concentration	Population	FST of participants	Follow-up duration	Outcomes	Complications
Huang et al., 2025 [17]	Single-center, double-blind, randomized study	Salicylic acid 1% and 2%	54 participants ranging from 18 to 45 years (49 females and 5 males)	25 participants had FST I-III. 29 participants had FST IV-VI	48 hours to 3 months	Reduced acne severity and skin oiliness. Evens out skin tone without overdrying or irritating the skin	None reported, potential drying of skin
Veenstra et al., 2021 [18]	Case study	Trichloroacetic acid 100%	1 46-year-old male	FST V	2 months	Improved acne scars	Application with a micropipette led to necrosis of adjacent healthy skin
Syder et al., 2025 [19]	Multisite, retrospective chart review study	Azelaic acid and salicylic acid (concentrations not specified)	1,355 participants ranging from 15 to 78 years (888 females and 467 males)	Not mentioned	Not mentioned	Chemical peels prescribed slightly more in White patients; orals prescribed more in Black patients	Disparity of prescription in patients of color leads to the undertreatment of acne
Kulthanan et al., 2020 [20]	Double-blind, randomized, vehicle-controlled study	Licochalcone A, 1,2-decanediol, L-carnitine, and salicylic acid 1%	110 participants ranging from 18 years old and above. Male and female participants not specified	Not mentioned	3 months	Significant reduction in the mean counts of noninflammatory, inflammatory, and total lesions	No complications
Rullan et al., 2020 [21]	Retrospective chart review	Carbolic acid 88%	139 participants ranging from 18 years old and above. Male and female participants not specified	50 participants had FST I-III. 89 participants had FST IV-VI	7 months	The triple approach to treating acne scars resulted in consistently high satisfaction among patients and photographic evidence of improvements	Tiny scabs and skin peeling
Wolff et al., 2016 [22]	Case study	Glycolic acid 35%, 50%, and 70% and Jessner's solution	1 18-year-old male	Not mentioned	4 months	Skin findings responded dramatically to a combined regimen of daily topical azelaic acid foam and tretinoin cream with twice-monthly chemical peels	Dyschromia on the arms did not improve as much as the face
Han et al., 2019 [23]	Case study	Trichloroacetic acid 30% and unknown herbal substances	1 25-year-old male	Not mentioned	2 years	Severe atrophic scars with occasional hypo- and hyperpigmentation	Deep second‐degree chemical burns on over 30% of the face

Chemical Peel Routine 

A salicylic acid-based regimen demonstrated comparable efficacy to standard prescription retinoid therapy, showing early and sustained improvements in acne severity, lesion counts, oiliness, and post-inflammatory changes while producing fewer side effects and achieving high patient satisfaction [17]. Another study investigating TCA application with a precise applicator, such as a 30-gauge needle, found it superior to electronic micropipette delivery, which carried a higher risk of tissue necrosis and scar worsening [18]. Chemical peels, including azelaic and salicylic acids, were more likely to be prescribed in White patients, while oral medications and antibiotics were more likely to be prescribed in non-White (Black or Hispanic) patients, despite similar or increased severity of acne, highlighting treatment inequities [19]. For maintenance therapy, a moisturizer containing licochalcone A, 1,2-decanediol, L-carnitine, and salicylic acid significantly reduced total lesions; however, there was no significant difference in skin dryness, stinging/burning, or pruritus between the treatment and control group [20]. Finally, another study used a triple approach to treatment, which included chemical reconstruction of skin scars using carbolic acid, subcision, and microneedling, and proved to have high satisfaction among patients and photographic evidence of improvements [21]. Another article reporting a case study of lichen planus pigmentosus showed significant facial improvement with daily topical azelaic acid foam and tretinoin cream and twice-monthly chemical peels using glycolic acid for the face and Jessner's solution for arms. At 16 weeks, facial dyspigmentation had markedly improved, while arm lesions showed mild changes [22]. While beneficial results were seen from most studies, one particular case study reported chemical burns after using a 30% TCA chemical peel with unknown herbal substances, which caused severe burning and wide facial ulcers [23].

Efficacy of Chemical Peels 

Overall, five out of the seven studies analyzed indicated that chemical peels were helpful in improving acne scars and skin dyspigmentation. Minimal side effects were found, the most common being small scabs and peeling or dryness of skin. A study reported by Huang et al. showed that there were significant improvements (p≤0.001) in the mean Investigator's Global Assessment of acne severity from 48 hours through 12 weeks and significant improvements from baseline in total acne lesion count, global PIH/erythema, and oiliness [17]. Additionally, it was found that in this particular study, the salicylic acid chemical peel was well tolerated as the mean score was below mild for all parameters, whereas the control prescription group caused significantly more tightness/dry feeling at the fourth-week measurements (p=0.008) [17]. Subjects (>96%) also reported high satisfaction using the chemical peel at all time points [17]. Overall, these and similar positive findings highlight the effectiveness and promising results of chemical peels, particularly in skin of color.

Discussion

This scoping review demonstrates that chemical peels, when applied with proper technique and patient selection, can be both safe and effective for individuals with skin of color [24]. Across the included studies, the most frequent indications were acne and PIH, conditions that disproportionately affect patients with darker FST [16,25]. These findings suggest that chemical peels may offer a valuable therapeutic option in skin of color dermatology, particularly for disorders where pigment alteration is central to disease burden.

Limitations of the Study

Despite these encouraging outcomes, significant limitations were noted in the available evidence. Study populations lacked diversity, often underrepresenting certain ethnic groups within the broader category of skin of color [26]. In addition, most research focused on a narrow range of peeling agents, primarily glycolic and salicylic acid, while other potentially useful peels, such as TCA or combination protocols, were less frequently studied [27,28]. This restricts the generalizability of existing data and underscores the need for more inclusive and methodologically robust studies.

Research Implications of the Study

When compared to existing literature and clinical guidelines, our findings align with prior reviews suggesting that superficial peels can be used safely in patients with FST IV-VI if performed cautiously [4,14,29]. However, unlike larger systematic reviews that include predominantly lighter skin types, our focused analysis highlights unique challenges in darker phototypes, particularly the elevated risk of PIH and scarring [25]. These differences emphasize the importance of tailoring chemical peel protocols specifically to patients with skin of color, rather than extrapolating from data in lighter-skinned populations.

This review highlights several important considerations for clinicians managing patients with skin of color. Superficial chemical peels, particularly glycolic and salicylic acid, can be used safely when conservative concentrations, gradual escalation, and careful patient selection are employed [4,14]. Pre-procedural optimization, including control of active inflammatory conditions such as acne or melasma and strict post-procedural photoprotection, is essential to minimize the risk of PIH [9,24]. Clinicians should favor agents with more predictable penetration profiles, such as mandelic acid, in higher FST [4,14,23] and exercise caution with medium-to-deep peels, including higher-strength TCA formulations, due to the increased risk of pigmentary alteration and scarring [7,26]. Finally, practitioner familiarity with skin of color dermatology and individualized treatment planning remains critical to achieving safe and effective outcomes [15,24,26].

Practical Implications of the Study

From a clinical standpoint, this review underscores the importance of individualized treatment planning based on both the patient's underlying dermatologic condition and their FST [15]. Practitioner training in skin of color dermatology is essential to minimize complications and optimize outcomes [24]. Furthermore, standardized guidelines addressing appropriate peel selection, pre- and post-procedure regimens, and long-term outcome monitoring are needed to improve both safety and efficacy [26].

Future Directions

In summary, while current evidence supports the cautious use of chemical peels in patients with skin of color, there remain important gaps in knowledge. Expanding the diversity of study populations, testing a broader range of peeling agents, and developing practitioner education programs tailored to skin of color will be critical next steps in advancing equitable and evidence-based care. 

TCA Peels for Melasma

Furthermore, upon investigating the gaps and limitations of chemical peels pertaining to skin of color, there are only a few studies that address the safety concerns and efficacy of using TCA and glycolic acid peels. The review on the treatment of melasma using TCA peels in Latin American women displayed a small sample size with only 33 patients completing the study across a single randomized, prospective trial [30]. The results gathered from this trial remain limited due to the fact that there were no controls, only one blinding assessment tool, and a small sample size, to name a few [30]. Another limitation contributing to the small selection of the study design was the lack of clarity when it came to the darker phototypes V and VI. In this particular study, only FST III and IV were selected in effectively treating melasma by the use of TCA peels in combination with tretinoin and hydroquinone [30]. Therefore, there is not enough evidence to determine if these procedures are safe on skin with a greater concentration of melanin. 

Chemical Peel Effects in FST V

On another note, in a retrospective cross-sectional monocentric review investigating the safety and effects of high levels of glycolic acid and TCA peels in darker skin phototypes, greater attention was drawn to FST V specifically [31]. According to a single-center review, modified melasma area and severity index scores at 12 weeks demonstrated significant improvement in melasma cases across a majority female population seeking treatment [31]. Notable adverse effects evident among individuals of FST V were irritation in 77.5%, postpeel cracking in 62.5%, and transient hyperpigmentation in 12.5% [31]. Melasma recurred in about 70% of these women after 12 weeks of discontinuing all chemical peel treatments [31]. Although there were recurrences, glycolic acid and TCA were both effective in reducing melasma without resulting in scarring [31]. However, since there were no known cases of patients with skin type VI receiving treatment by way of these chemical peels and a lack of randomized controlled trials as well as a lack of long-term data beyond 12 weeks, further findings must be collected to address the safety, efficacy, and practice patterns of commonly used chemical peels such as glycolic acid and TCA in darker skin phototypes of color. 

Mechanisms of PIH and Pigmentary Disorders

In order to understand the long-term effects of PIH, it is important to understand the mechanism of action in skin of color. According to a study on PIH in dark skin, melanin absorbs and reflects ultraviolet radiation (UVR), preventing damage to genomic DNA in the epidermis and degradation of collagen in the dermis [32]. However, increased content of melanin in combination with the extrinsic stress factors causing inflammation, such as excess UVR, allergic reactions, or injury, can also frequently lead to cosmetic problems resulting in discoloration and scarring [32]. Knowing this allows clinicians to take a more thorough and cautious approach when faced with patient cases that bring up concerns with PIH related to chemical peels in skin of color. Additionally, some findings suggest that acne-induced hyperpigmentation can significantly impact the quality of life in individuals with skin of color [33]. Pigmentary disorders such as melasma and acne-induced hyperpigmentation require cautious treatment to avoid exacerbation, while the unregulated use of skin bleaching agents containing high-potency corticosteroids can pose serious health risks [33]. This further emphasizes the importance of evaluating whether or not a patient is an appropriate candidate for a chemical peel based on their current medical history by acknowledging if there is pre-existing or existing inflammatory acne or another known condition such as melasma. By addressing these concerns in a consultation visit, there is less of a chance of worsening these conditions. A chemical peel may risk causing more of an issue in these patients with pigmentary disorders who have skin of color. 

Biologic Differences in Skin of Color Relevant to Chemical Peels

Biologic differences in skin of color are a major factor in both the safety profile of chemical peels and their clinical results. While the density of melanocytes is more or less comparable in all skin types, FST IV-VI show aided melanocyte transformation. Besides that, their melanosomes are larger and more dispersed than those of the lighter skin types; this altogether makes them more likely to suffer from PIH after epidermal injury has occurred [9,24]. Since the epidermis is purposefully broken by the peel, even minor inflammation can lead to long-lasting pigmentary changes in these patients.

Response to treatment is also influenced by the presence of different skin types. Patients with darker skin have thicker epidermis, more dense stratum corneum, and differently organized collagen in their dermis; all these characteristics can modify the penetration and healing dynamics of the peel [13,14]. If not controlled properly, these traits may cause unpredictable depth of injury due to excessive strength or prolonged time of the peel used. Moreover, increased fibroblast activity and collagen reactivity may be among the reasons why maturing or deep peels on these skin types come with a higher risk of hypertrophic scarring or keloid formation [12].

These biological traits underline the necessity to resort to very cautious peel selection, advanced methodology, and proper pre- and post-procedural care to curb inflammation and avoid pigmentary sequelae in the treatment of patients with skin of color.

Long-Term Pigmentary Risk and Clinical Considerations

While short-term outcomes of chemical peels in patients with skin of color are generally favorable when the appropriate technique and patient selection are employed, long-term pigmentary complications warrant careful consideration. In particular, deeper chemical peels and higher concentrations of TCA, as well as phenol-based peels, have been associated with an increased risk of persistent hypopigmentation, especially in individuals with higher FST [7,26,33]. This risk is thought to result from melanocyte injury or prolonged suppression of melanogenesis following deeper epidermal or dermal damage, emphasizing the importance of limiting peel depth and concentration in this population [26,33].

Photoprotection before and after chemical peeling is a critical component of standard of care in patients with skin of color, as UVR can exacerbate both PIH and hypopigmentation [9,24,33]. Strict sun avoidance, consistent use of broad-spectrum sunscreen, and comprehensive patient education regarding adherence to post-procedure photoprotection protocols are essential to minimizing pigmentary sequelae and optimizing long-term outcomes [9,26].

Emerging evidence also supports the increasing use of mandelic acid peels in patients with skin of color due to their larger molecular size, slower epidermal penetration, and more favorable safety profile compared with other alpha-hydroxy acids [4,5,14]. These characteristics may reduce the risk of melanocyte injury and dyschromia, making mandelic acid a promising alternative for individuals at higher risk of pigmentary complications, particularly those being treated for acne or PIH [4,14,23].

## Conclusions

Patients with skin of color can benefit from chemical peels when practitioners select appropriate peel types and depths while assessing individual risk variables. Superficial agents, including glycolic and salicylic acid, show promising results in treating acne and PIH as well as certain pigmentary disorders while maintaining good safety records. The generalizability of research findings is limited since multiple studies include a small sample size and brief observation periods, along with insufficient representation of FST IV-VI subjects. Deeper peels, along with TCA and phenol agents, have insufficient data regarding their use in darker skin phototypes, and multiple knowledge gaps exist in standardized treatment protocols, ethnic subgroup representation, and socioeconomic accessibility. Future research should focus on conducting larger clinical trials with diverse patient groups. It should also study additional peeling agents and combination protocols alongside developing educational material specifically for practitioners who treat skin of color. Research advances will enable chemical peels to become a safer, more predictable, and equitable treatment choice for diverse patient populations.
